# Microcystis Chemotype Diversity in the Alimentary Tract of Bigheaded Carp

**DOI:** 10.3390/toxins11050288

**Published:** 2019-05-22

**Authors:** Milán Riba, Attila Kiss-Szikszai, Sándor Gonda, Gergely Boros, Zoltán Vitál, Andrea Kériné Borsodi, Gergely Krett, Gábor Borics, Andrea Zsuzsanna Ujvárosi, Gábor Vasas

**Affiliations:** 1 Department of Botany, Institute of Biology and Ecology, Faculty of Science and Technology, University of Debrecen, H-4032 Debrecen, Hungary; milan.riba@gmail.com (M.R.); gondasandor@gmail.com (S.G.); ujvarosiandi@gmail.com (A.Z.U.); 2 Department of Organic Chemistry, Institute of Chemistry, Faculty of Science and Technology, University of Debrecen, H-4032 Debrecen, Hungary; kiss.attila@science.unideb.hu; 3 Balaton Limnological Institute, MTA Centre for Ecological Research, H-8237 Tihany, Hungary; boros.gergely@okologia.mta.hu (G.B.); vital.zoltan@okologia.mta.hu (Z.V.); 4 Department of Microbiology, ELTE Eötvös Loránd University, H-1117 Budapest, Hungary; borsodi.andrea@ttk.elte.hu (A.K.B.); krett.gergely@okologia.mta.hu (G.K.); 5 Danube Research Institute, MTA Centre for Ecological Research, H-1113 Budapest, Hungary; boricsg@gmail.com

**Keywords:** *Microcystis*, chemotype, bigheaded carp, anabaenopeptin, microginin, microcystin

## Abstract

Most cyanobacterial organisms included in the genus *Microcystis* can produce a wide repertoire of secondary metabolites. In the mid-2010s, summer cyanobacterial blooms of *Microcystis* sp. occurred regularly in Lake Balaton. During this period, we investigated how the alimentary tract of filter-feeding bigheaded carps could deliver different chemotypes of viable cyanobacteria with specific peptide patterns. Twenty-five *Microcystis* strains were isolated from pelagic plankton samples (14 samples) and the hindguts of bigheaded carp (11 samples), and three bloom samples were collected from the scums of cyanobacterial blooms. An LC-MS/MS-based untargeted approach was used to analyze peptide patterns, which identified 36 anabaenopeptin, 17 microginin, and 13 microcystin variants. Heat map clustering visualization was used to compare the identified chemotypes. A lack of separation was observed in peptide patterns of *Microcystis* that originated from hindguts, water samples, and bloom-samples. Except for 13 peptides, all other congeners were detected from the viable and cultivated chemotypes of bigheaded carp. This finding suggests that the alimentary tract of bigheaded carps is not simply an extreme habitat, but may also supply the cyanobacterial strains that represent the pelagic chemotypes.

## 1. Introduction

Most cyanobacterial species can produce a wide range of secondary metabolites with diversified biological activities. The unique cyanobacterium-specific secondary metabolites originating from variable biosynthetic pathways show great chemical diversity and are common across cyanobacterial taxa. Many of these compounds that are of interest in several scientific fields (pharmacology, toxicology, ecology, etc.) have been isolated from strains cultured under controlled conditions and field samples, and partly or fully characterized [1,2].

Oligopeptides are a major family of cyanobacterial secondary metabolites. They are a highly diverse category of low molecular weight peptides built from proteinogenic and non-proteinogenic amino acids. The most widely accepted classification by Welker and von Döhren [3] is as follows: aeruginosins [4], cyanopeptolins [5], anabaenopeptins [6], microginins [7], microviridins [8], cyclamides [9], and the well-studied and notorious microcystins [10]. These oligopeptides are mainly synthesized by non-ribosomal pathways, assembled by large multifunctional enzyme complexes, regularly non-ribosomal peptide synthases (NRPS), or hybrid NRPS/PKS (polyketide) synthases. These complexes are encoded in large gene clusters with a modular unit, and produce mainly small peptide chains as end-products [11,12].

Oligopeptides are appropriate biomarkers of cyanobacterial subpopulations. LC-MS-based untargeted peptide metabolomic studies can be useful for the separation and identification of unknown metabolites from complex natural matrices, and for metabolite typing at both individual and population levels [13,14].

*Microcystis* is one of the most widely studied cyanobacterial genera due to its ability to form toxic blooms in freshwater environments across almost all continents. *Microcystis* blooms have increased in general during recent decades, and are expected to further expand in the near future. Furthermore, several cases of huge biomass production have occurred, which were linked to this genus in strategically important freshwater habitats [10]. High genetic diversity and genotype numbers have been identified in the genus *Microcystis* [15,16,17,18,19,20,21,22,23]. It is important to note that the reported existence of a number of described morphospecies based on colony/cell morphology is not supported by molecular evidence, forming a clade of nearly identical 16S rDNA sequences [24,25]. Based on the low 16S rRNA sequence variability and DNA–DNA hybridization data, Otsuka et al. [26] suggested merging all morphospecies into a single species following the Bacteriological Code rules.

Climate change and nutrient over-enrichment in waters has led to worldwide proliferation of various geno- and chemo-types of several cyanobacterial species [27]. Although the invasion of microorganisms to new aquatic environments is difficult to observe, several cyanobacteria have shown characteristic microscopic morphological features, or have generated conspicuous negative impacts on the local ecosystem [28,29]. Invasions may also threaten global biodiversity by changing the structure and function of ecosystems and interrupting key biological interactions [30]. Indeed, when invading new areas, cyanobacterial species (including *Microcystis* spp.) are able to cause fatal environmental changes by defeating native species, disturbing food-web structures [31], or reducing diversity [28,32,33].

Bighead carp (*Hypophthalmichthys nobilis* R.) and silver carp (*H. molitrix* V.) are filter feeder cyprinid fish, native to the large freshwater habitats of Asia [34,35]. These species and their hybrids (collectively referred to as filter-feeding Asian or bigheaded carps) are detritivorous, planktivorous, and opportunistic feeders [35,36]. They have been introduced into lakes, rivers, and reservoirs throughout the world since the early 1950s. The usual purpose of introducing and stocking bigheaded carp outside their native range is to increase fishery yields and improve water quality, because it is assumed that that these fish species (especially silver carp) are effective biological control agents for algal blooms [37,38,39,40]. Several promising biomanipulation experiments have been conducted and found that population size and quantity of cyanobacteria were unchanged or even increased by stocking filter-feeding fish [41]. In addition, filter-feeding fish might increase nutrient availability and could thus stimulate the proliferation of cyanobacteria [42,43]. Miura and Wang [44] noted that several cyanobacteria survived after passing through the gut of filter-feeding species, and attained increased photosynthetic activity. Many studies conducted on bigheaded carps reported no negative effects on the viability of some cyanobacterial species after gut passage [45]. Lewin et al. [46] and Görgényi et al. [47] proposed that *Microcystis* cells were not harmed or damaged after transit through the gut due to their mucous protection. Moreover, direct use of phosphorus by this cyanobacterial species has been detected in fish guts during passage [47].

**Figure 1:**
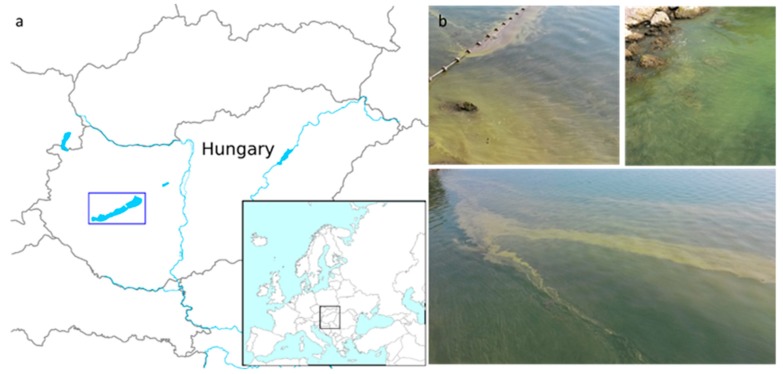
(**a**) Location of Lake Balaton in Hungary and Central Europe, indicated by a blue rectangle. (**b**) The *Microcystis* bloom in the lake (August 2015).

**Figure 2:**
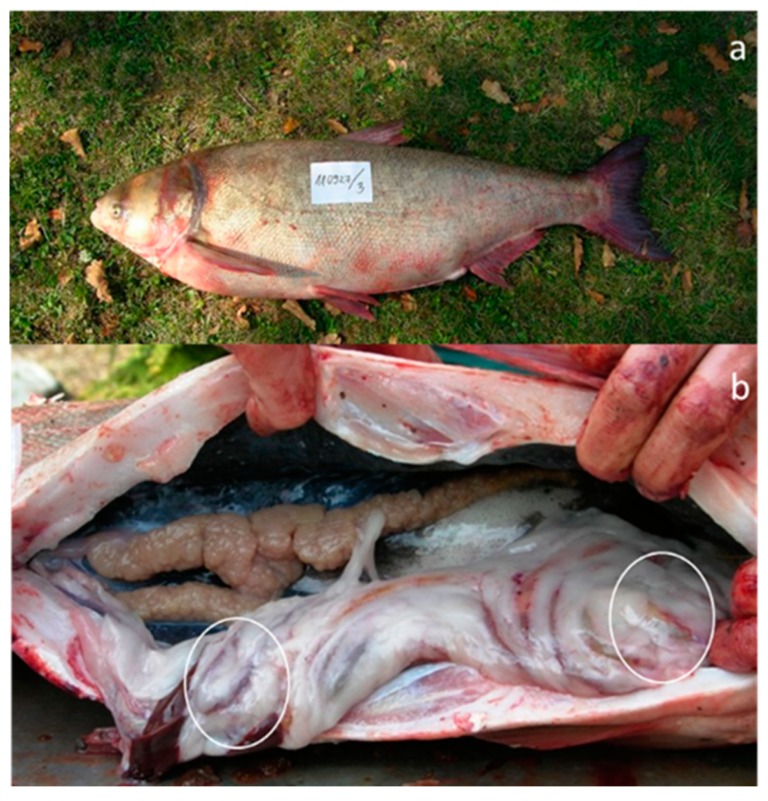
(**a**) A hybrid bigheaded carp (total body length 1.08 m; weight 23 kg) caught in Lake Balaton, Hungary. (**b**) A dissected male bigheaded carp, showing the testicles and the guts in the abdominal cavity. Foregut (to the right side of the image) and hindgut (to the left) are denoted by white ellipses.

In the mid-2010s, summer cyanobacterial blooms of *Microcystis* spp. occurred on a regular basis in Lake Balaton, the largest lake in Central Europe (Figure 1). The main objective of the present work was to investigate *Microcystis* chemotypes within these waterbodies in the period between 2013–2016. More specifically, we aimed to: (i) Identify new and well known congeners of the cyanobacteria peptide family using a LC-MS-based untargeted approach; and (ii) determine the pattern, abundance, and distribution of *Microcystis* chemotypes among the pelagial, bloom area, and in the alimentary tracts of filter-feeding bigheaded carps (Figure 2)—as this fish species represent a massive stock in the lake recently.

## 2. Results

### 2.1. Morphology-Based Identification of Cyanobacterial Bloom Causing Organisms, and the Isolated Strains

*Microcystis* cells were spherical with a 5–8 µm diameter. Aggregated cells were organized into colonies with very narrow and diffluent colorless mucilage. Light blue-green protoplasts appeared to be light-brown due to the optical effects of gas vesicles which were observed in the cells. Colony sizes ranged from microscopic to macroscopic. The macroscopic colony layer showed a pale green color in the natural blooms and cultures. Morphological features were identical to the characteristics of *Microcystis flos-aquae* [48]. Based on morphological criteria of the colinies, the dominant morphotype in the bloom samples was identified as *Microcystis flos-aquae*.

### 2.2. Molecular Phylogenetic Analyses

**Figure A1:**
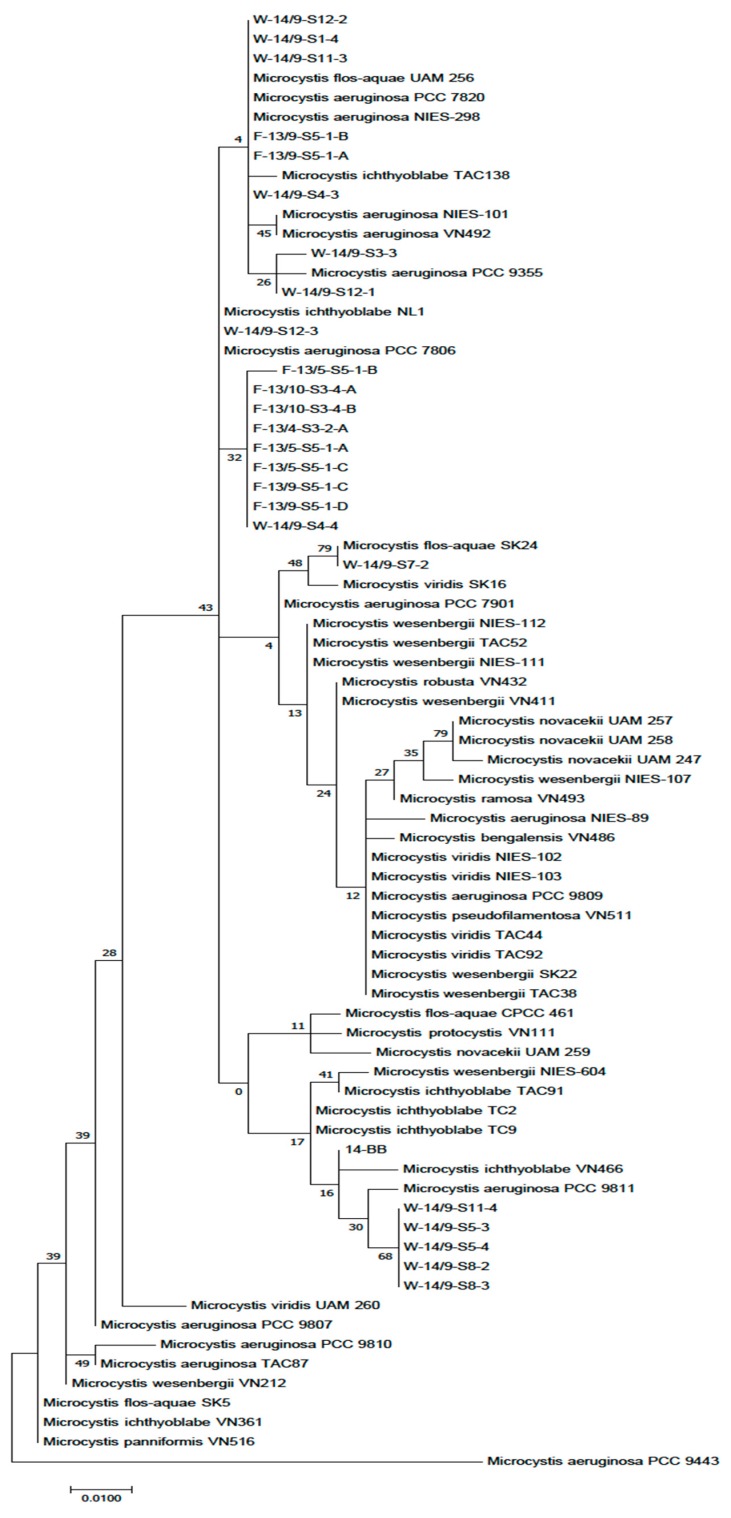
Phylogenetic dendrogram of the studied *Microcystis* strains. The dendrogram was based on the 16S–23S internal transcribed spacer region sequences and the evolutionary history was inferred using the maximum likelihood method based on the Jukes–Cantor model.

In the phylogenetic analysis, altogether 25 ITS strain sequences were isolated from Lake Balaton and hindgut content of bigheaded carps (*Hypophthalmichthys* spp.). These were involved with *Microcystis* ITS sequences originating from six different morphospecies. All ITS sequences in this study formed a distantly related cluster with all but one *Microcystis* morphospecies (Figure A1). In the phylogenetic tree, the ITS sequences of strains from the lake’s water and hindgut samples were arranged into eight separated lineages, however, no clear distinction according to sample type or sampling time could be observed. Our strains showed the closest phylogenetic relation to morphospecies *M. flos-aquae* and *M. viridis*.

### 2.3. Identification of Peptides and Comparative Analysis of Bloom Samples and *M. flos-aquae* Strains

The cyanobacterial peptides identified from *M. flos-aquae* strains and blooms that originated from the pelagic and bigheaded carp matrix are presented in Table 1, Table 2 and Table 3.

Seventeen variants from the microginin (MG) class were present in our samples (Table 1). MG FR3 (*m/z* 728 [M + H]^+^) and FR4 (*m/z* 742 [M + H]^+^) were identified from the MS^2^ data [49]. MGs with *m/z* 565 [M + H]^+^ and *m/z* 579 [M + H]^+^ showed identical fragmentation patterns but in both cases, the last tyrosine unit was absent. The 16 Da mass difference in the MS dataset of *m/z* 581 [M + H]^+^ and *m/z* 744 [M + H]^+^ indicated the presence of an additional hydroxyl group on tyrosine at position 4 in these congeners. MG *m/z* 754 [M + H]^+^ contained amino acid modification in two positions compared to FR3. The 26 Da mass increase can be explained by an amino acid exchange from threonine to leucine at position 2, and a substitution of tyrosine with homotyrosine at position 4, which is also supported by MS^2^ data (Table A1).

The structure of MG *m/z* 770 [M + H]^+^ was deduced from the amino acid sequence of MG 478 [50] with the same molecular weight. However, Ahda and leucine amino acids were at positions 1 and 2, respectively, as concluded from the 127 Da mass difference between the product peaks and presence of a fragment with 299 *m/z*. MGs with *m/z* 784 [M + H]^+^ and 798 [M + H]^+^ were identified as methylated analogs of MG 770 with N-methyl-Ahda at position 1 in both cases and an amino acid exchanged from methyl-valine to methyl-leucine at position 2 for the latter. MG *m/z* 607 [M + H]^+^ and MG *m/z* 621[M + H]^+^ were characterized as shortened forms of MG 770 and 784, composed of four amino acid units. MG *m/z* 591 [M + H]^+^ was found to be an analog of MG 621, and based on its fragmentation pattern (the 30 Da neutral loss affected only position 4), this variant contains phenylalanine at position 4 (Table A1).

MG *m/z* 712 [M + H]^+^ was identified as a methylated form of MG T2, whose modification affects proline at position 4 according to the MS^2^ data. MG *m/z* 549 [M + H]^+^ and *m/z* 595 [M + H]^+^ were found to be shortened variants of methylated-T2 with tyrosine and hydroxy-tyrosine at position 4. The structure of MG *m/z* 579 [M + H]^+^ was derived from MG *m/z* 549 [M + H]^+^ with an amino acid substitution from alanine to threonine at position 2 (Table A1).

Fifteen completely characterized—and an additional 21 partially characterized—anabaenopeptin (ANA) variants were identified in our samples. ANA A (*m/z* 844 [M + H]^+^) and B (*m/z* 837 [M + H]^+^) showed the same MS^2^ and MS^3^ fragmentation pattern as previously reported by Mayumi [51]. Other previously known variants include oscillamide (OSC) Y (*m/z* 858 [M + H]^+^) and ANA F (*m/z* 851 [M + H]^+^). Although ANA F and E variants had the same molecular mass, the two ring structures differed only in their MS^3^ spectra. In our case, MS^3^ product ions supported only the structure of ANA F (Table A2). ANAs with *m/z* 828 [M + H]^+^ and *m/z* 842 [M + H]^+^ as well as *m/z* 916 [M + H]^+^ and *m/z* 930 [M + H]^+^ showed the same MS^3^ fragmentation pattern as ANA A and OSC Y. For the first pair, the 16 Da neutral loss indicated the presence of homophenylalanine in position 4 instead of homotyrosine, which was also supported by MS^3^ data. In the second pair, the difference in molecular mass of 72 Da affected two positions. According to the MS^3^ information, a 106 Da mass increase at position 5 indicated a substitution from N-methyl-alanine to N-methyl-homotyrosine, and a 34 Da mass reduction at position 6 suggested a change from phenylalanine to isoleucine. Seven completely characterized variants showed unusual product ions in the MS^2^ experiments. The 26 Da mass difference between the two product ions was linked to the presence of the CO-group between the ring and the side chain, but these products indicated an amino acid with a 209 Da mass in position 1, which could be explained by the presence of a tyrosine derivative like *N*-methyl-homotyrosine. Among these congeners, ANA *m/z* 872 [M + H]^+^ and *m/z* 886 [M + H]^+^ showed identical structures to ANA A and OSC Y. In the case of ANA *m/z* 852 [M + H]^+^ and *m/z* 866 [M + H]^+^, the 20 Da mass loss indicated methyl-leucine at position 6, while the 14 Da mass increase of ANA *m/z* 900 [M + H]^+^ suggested methyl-phenylalanine at position 6. ANA *m/z* 856 [M + H]^+^ and *m/z* 870 [M + H]^+^ have homophenylalanine at position 5 deduced from the loss of 16 Da (Table A2).

Each of the partially characterized ANAs showed characteristic product ions with the 26 Da difference in the MS^2^ experiences. In four cases, no amino acid or a simple derivative thereof could be assigned to amino acid position 1 based on MS/MS, but their ring structure suggested that these were compounds related to previously known ANAs. For the remaining cases, the MS analysis data were insufficient to determine all the structural elements due to the low peak intensity (Table A2). 

In our samples, 13 microcystin (MCY) variants were identified (Table 3). MCY-RR and -WR were found in a double protonated form with *m/z* 520 [M + 2H]^2+^ and *m/z* 513 [M + 2H]^2+^ [52]. The MCY-LR, -FR, -YR, and -WR forms were singly-charged and gave protonated ions at *m/z* 995 [M + H]^+^ and *m/z* 1029 [M + H]^+^, respectively. Modification at position 7 was observed in four cases. Peaks with *m/z* 1015 [M + H]^+^ and *m/z* 1031 [M + H]^+^ were identified as [Dha7]MCY-FR and [Dha7]MCY-YR, respectively. Furthermore, *m/z* 1014 [M + H]^+^ and *m/z* 1086 [M + H]^+^ were evaluated as [MeSer7]MCY-LR and [MeSer7]MCY-WR, respectively. A non-methylated asparagine was present at position 3 in [D-Asp3]MCY-LR, which gave *m/z* 981 [M + H]^+^. Non-proteinogenic amino acids were found at position 2 in two cases. A peak with *m/z* 1009 [M + H]^+^ was found to be a homoisoleucine-containing analogue (MCY-HilR), and *m/z* 1049 [M + H]^+^ was identified as MCY-(H4)YR [52,53] (Table A3). 

The heatmap shows the relative abundance of given natural products in the *Microcystis* samples—the color is proportional to the log10 of the area under the curve values.

**Figure 3:**
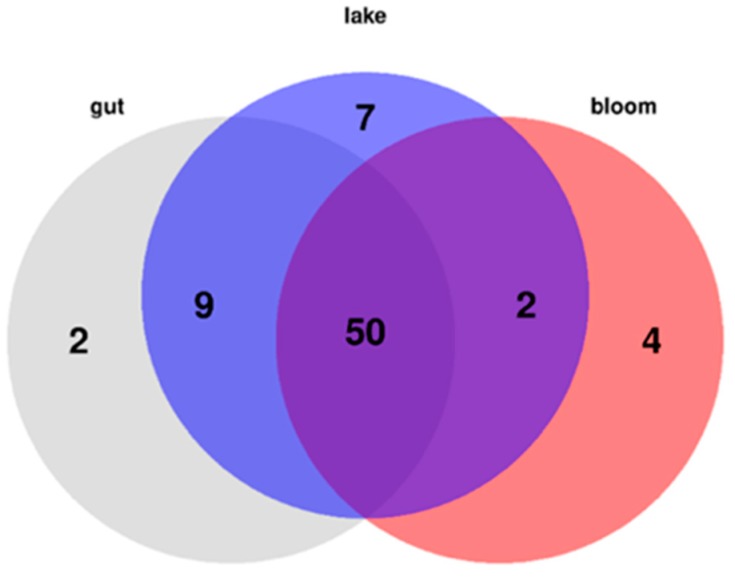
Venn diagram showing the presence/absence of identified peptide natural products in the dataset containing the gut, bloom, and lake samples. A single sample was enough to state that the group contained the metabolite of interest.

**Figure 4:**
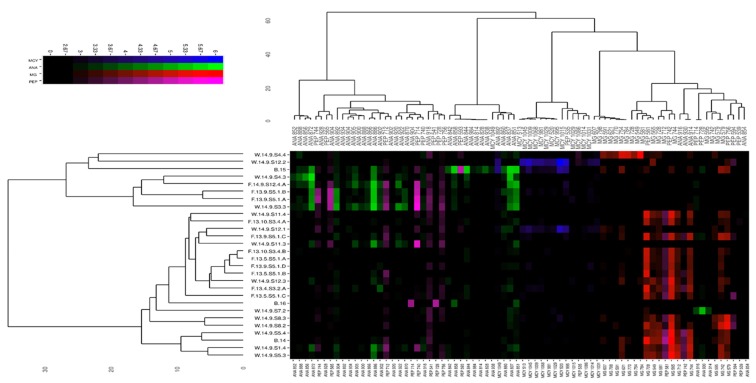
Heatmap of the abundance values for peptide natural products from the bloom, water, and gut samples. Compounds and samples were sorted along the axes after hierarchical clustering in R 3.5.0. Color is proportional to log10(abundance) of the compounds, as obtained by LC-ESI-MS measurements, and indicated in the color gradient legend. The different peptide subclasses were mapped to different colors as follows: microcystins (MCY) are shown in blue; anabenopeptines (ANA) are shown in green; microginins (MG) are shown in red; other peptides (PEP) are shown in magenta. The *x*-axis contains labels showing metabolite class and *m/z* of [M + H]^+^. The *y*-axis labels show sample IDs. The presence of certain chemotypes (composed of isolates from gut, lake, and bloom samples) can be easily recognized.

The most important phenomenon is the lack of peptide pattern separation for *Microcystis* originating from the gut, lake, and bloom-samples. The samples were separated into relatively loose clusters based on their metabolomes, spreading all three functional groups throughout these clusters. No separation by sampling year could be found either (Figure 3 and Figure 4).

### 2.4. Classification of Oligopeptide Pattern into Chemotypes

**Figure 5:**
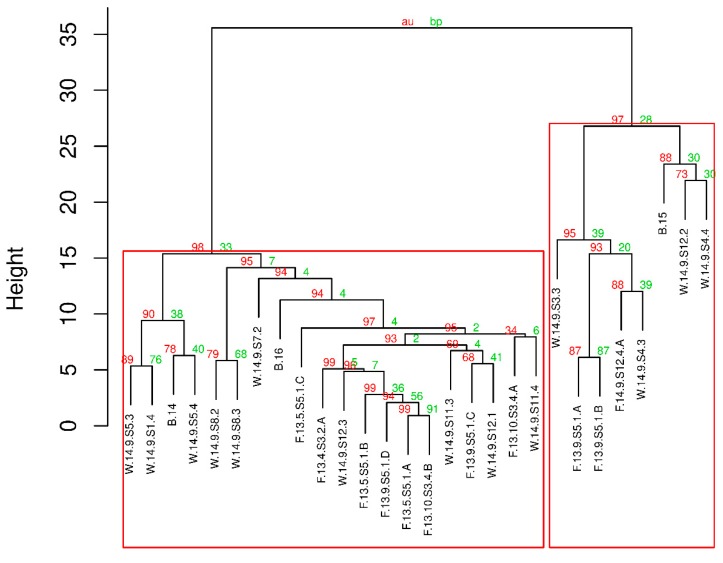
Bootstrapped hierarchical clustering of algal lines according to scaled and centered natural compound abundance values, using the Minkowski distance and Ward’s hierarchical clustering, bootstrapping *N* = 1 × 10^6^. The clusters highlighted in red rectangles exist at a *p*-value < 0.05.

Investigating individual *Microcystis* strains and samples showed a high number of different, but more or less distinct, chemotypes (with different peptide patterns) that originated from all habitats. On the other hand, more or less distinguishable chemotypes were observed among the samples, which were separated into clusters of varied densities based on the absence or co-occurrence of biosynthetic metabolite clusters. The samples either contained: (1) a variety of ANAs and a few unidentified peptides, (2) MCYs, or (3) MGs as their major compounds, and usually contained significantly lower or no detectable amounts from other peptide groups. For example, in many samples, a group containing ANAs that lack MGs and MCYs were seen to be of this chemotype. The co-occurrence of several MCY types was observed in only a few samples. This was also illustrated by the compact cluster separating MCYs from other natural products. These MCY-rich samples lacked other chief compounds of interest. Another subset of cyanobacteria contained various MGs at high concentrations, but did not contain ANAs or other peptides as major components. These compounds were all present in their producers, resulting in a relatively compact MG cluster. The bootstrapped hierarchical clustering analysis revealed the existence of two main chemotypes: MG-dominant and ANA-dominant (Figure 5). The existence for both clusters was *p* < 0.05.

### 2.5. Lack of Phylogenetic Relationship Was Found among the Chemotypes

Although all of the isolates were identified as the *M. flos-aquae* morphotype by conventional microscopy techniques, several phylogenetic clusters (Figure A1) and more distinct chemotypes (Figure 4 and Figure 5) have also been defined. The lack of a phylogenetic relationship was found among the chemotypes. To test the statistical significance of chemical patterns and other variables (such as origin, phylogenetic position), principal component score values were subjected to the Kruskal–Wallis significance test, where the variable studied was the group-determining factor. This approach for all metabolites allowed higher statistical power compared to direct statistical analyses. No significant association was found between the metabolite pattern, abundance, and genetic background. The 16S–23S ITS phylogenetical group had no significant effect on the metabolite pattern (*p* > 0.05), represented by one of the 28 PC dimensions in the analysis. No significant association was found between the metabolite pattern, abundance, and source of isolate. Isolate source (water, fish, or bloom) had no significant effect on the metabolite pattern (*p* > 0.05), represented by one of the 28 PC dimensions in the analysis.

## 3. Discussion

The detected *Microcystis flos-aquae* blooms were unexpected but not unique, because at the beginning of the 20th century, *Microcystis* blooms were observed in Lake Balaton localizing to small areas [54]. In addition, from the middle of the century, mainly nitrogen fixing filamentous cyanobacteria species like *Aphanizomenon* and *Anabaena* (*Dolichospermum*) caused this phenomenon in the lake. In Lake Balaton, the species *Cylindrospermopsis raciborskii* was identified in the 1970s and initiated whole-lake-area sized water blooms in 1982 and 1994, which adversely affected the tourism and economy of the area [55]. A number of comprehensive water management measures at the end of the 1980s were aimed at curbing eutrophication such as the drainage of communal sewage from the coastal zone. At the same time, the scale of agricultural activity decreased, resulting in lower nutrient loads to the lake. From this period until the present report, there has been no cyanobacterial blooming in Lake Balaton. The reappearance of *Microcystis* blooms in the lake, which were observed in this study, suggests that external nitrogen loads may have initiated the multiplication of non-diazotrophic cyanobacteria. In our present study, 11 *Microcystis* strains were isolated from gut samples, 14 from the pelagic plankton from Lake Balaton, and three collected *Microcystis* bloom samples.

Taxonomic classification of *Microcystis* is difficult. The combination of microscopic observations with molecular data can be the most adequate method for identifying isolates [56]. Most species of this genera have been described via their morphological characteristics [57,58], however, colony variance can be huge, and the external qualities of many populations overlap the limiting specifications [59,60]. Therefore, it is difficult to find the differences between traditional species [61].

The taxonomic position of the isolated strains was confirmed by phylogenetic analysis. Our strains showed the closest phylogenetic relation to morphospecies *M. flos-aquae* and *M. viridis* (Figure A1). Several papers have been published on the correlation between *Microcystis* morphotypes and genotypes [62,63]. The heterogeneity of this genus between different regions has also been well documented using further genetic markers [16,18,19,20,22,23,29]. Neilan et al. [64] defined genetic similarity and noted that the species had no specific phylogeographic structure, which was in accordance with work described by Bittencourt-Oliveira et al., (2001) [17] where *Microcystis* strains did not show any distinct phylogenetic pattern.

In our work, we identified a strong mucous envelope characteristic of the studied *Microcystis* morphotype. This can explain how this cyanobacterial species survive in the alimentary tract of bigheaded carps. Exopolysaccharides (EPS) can be crucial for cellular attachment, adhesion, and survival. This highly hydrated layer provides protection to cyanobacterial cells against desiccation, toxic agents, or the digestive enzymes of other organisms. This role of the EPS has been confirmed in several studies [65].

Thirty-six ANA and 17 MG variants were identified from the isolated strains (Table 1 and Table 2). New MCY congeners are more rarely identified, perhaps because this has been the most investigated cyanobacterial peptide family. However, bioactive peptide families like MGs and ANAs are receiving growing attention. Four known and 32—to the best of our knowledge—previously unknown ANA variants were fully or partially identified in our analysis. Several partly identified peptide fragments were detected (Table 4 and Table A4), which were clustered into this family using heat map analysis.

ANA F, OSC B, and C are inhibitors of protein phosphatases (PP). N-MeHty and the positively charged Arg are crucial parts of molecules relating to this activity [66]. ANAs were also found to be active toward proteinase enzymes such as trypsin, chymotrypsin, elastase, and carboxypeptidase A [8,67,68]. The relaxing activity of rat aortic preparations was detected by treatments with ANA B and ANA 906 [69]. ANA B and ANA F, the most frequently found ANAs, were shown to inhibit the growth of many *M. aeruginosa* strains by inducing the lytic cycle in cyanobacteria [70,71]. Taking into account the published effects of these metabolites, its ecological roles might be important [72].

Two known, and 15—to the best of our knowledge—previously unknown MG variants were identified in our analysis. Several partly identified peptid fragments were also detected as part of this family.

MGs are a 40-member group of linear and nonribosomal peptides, which have been detected and purified from several bloom-forming cyanobacteria isolates. The number of congeners is growing [2,3,7]. These are built by an α-hydroxy-β-amino derivate of decanoic or octanoic acid, which is rarely chlorinated at its terminal methyl group with three to five additional amino acids in the molecules [4,7,73,74]. MGs are zinc metalloprotease inhibitors (e.g., angiotensin-converting enzyme), and aminoproteinases that bind their α-hydroxy-β-amino residue to the zinc at the active site of the enzyme. Our findings indicate that these substances are important candidates for treating hypertension [72]. The patchy distribution of oligopeptide patterns in cyanobacterial populations enables classifying isolates into several oligopeptide-based chemotypes [14,75]. It is important to note that mainly ANA and MG dominant strains were detected from Lake Balaton in this study, but 10 strains from the alimentary tract were MCY producing.

The distinguishable chemotypes we found in our analysis were separated into clusters of varied density based on the absence or co-occurrence of biosynthetic metabolite clusters, similar to several other studies that have investigated *Microcystis* and other cyanobacterial peptide producers such as *Planktothrix* sp., *Nostoc* sp., etc. [14,75,76,77]. These genera often possess variable chemotypes with the ability to produce different peptide families in natural assemblages [3]. In the *Microcystis* isolates originating from natural populations, four chemotypes were characterized based on the fact that they contained a few or several main peptides, but in many cases, the appearance of several different peptides belonging to different biosynthetic clusters has been observed.

**Figure A2:**
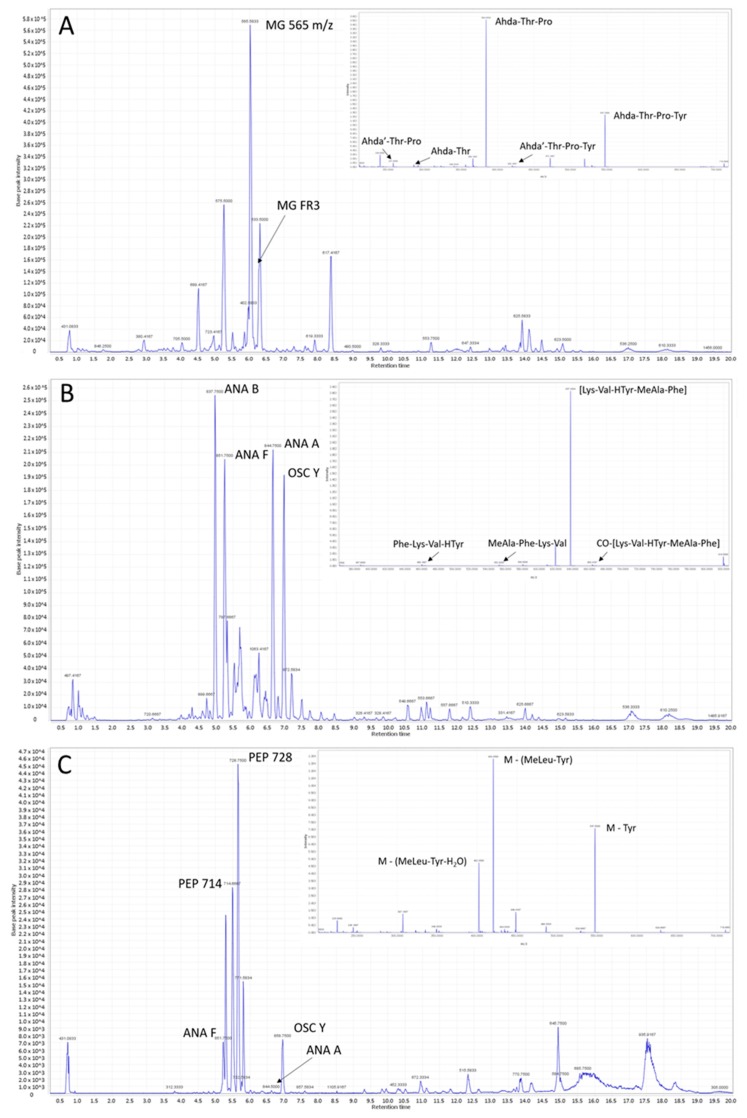
LC/MS chromatograms of the bloom samples 2014 (**A**); 2015 (**B**); 2016 (**C**); and the MS/MS fragmentation pattern of the most abundant peak.

From Lake Balaton, primarily ANA- and MG-dominated strains were detected, with the observation that many lesser-known or new congeners appeared from both groups of peptides. In addition, several partly identified peptide fragments were detected during the analysis whose metabolites seemed to belong to the metabolism (synthesis or degradation) of the two main groups identified as suggested by the heat map visualization. Focusing on the bloom samples from 2014–2016, it is important to note that the naturally collected material of all three samples represent mixed matrices, and each of them contained several chemotypes and/or genotypes of the genus *Microcystis*. These samples belonged to different chemotype clusters. The bloom samples from the same sampling site showed different bioactive peptide patterns. While the 2014 sample was mainly MG-dominated, the 2015 bloom sample was rather ANA-rich. The 2016 bloom community contained mostly non-identified peptides (Figure A2). Considering all the identified peptides in our samples, we found that the isolated and identified chemotypes originating from the gut and pelagic sample could be involved in the *Microcystis* community that built the bloom phenomenon.

Altogether, 13 MCY congeners were also identified in this work from phytoplankton and digestive tract strains. There is no doubt that MCYs are the most harmful and notorious family from the described cyanobacterial peptides. All of the detected MCY forms are already known, and no new MCY variant was identified in the present analysis. Although only a few MCY variants have been identified near the large number of MG and ANA, these peptide-producers display a separate cluster in our analysis. It is especially worth paying attention to this group because MCY is the most common toxin produced by cyanobacteria in waters [78,79], and can also cause death, illness, complications, and damage in humans, animals, and plant organs.

In the several cases where *Microcystis* and *Planktothrix* oligopeptide patterns have been investigated worldwide [12,13,14,75,80,81,82], and in our local area [83,84,85,86], the numbers of genotypes have been identified with the help of phylogenetic markers [64,87]. The lack of correlation between the *Microcystis* chemotypes and phylogenetic genotypes found in cases similar to our present study suggest that the synthesis of bioactive peptides is not phylogenetically conserved in this genera. This has also been the conclusion of recent work [88] where *Microcystis* chemotypes were researched in Spanish freshwater and reservoirs. The findings in our study are consistent with the statement that the distribution of oligopeptide production abilities does not correlate with morphospecies, phylogenies based on commonly used molecular markers, or the geographical origin of the isolated organisms [64,89].

Bigheaded carps were introduced into Lake Balaton (Hungary) in 1972 and were stocked until 1983 [90,91,92,93,94]. These filter-feeder fish species can consume almost all algal and cyanobacterial taxa from ambient water, but the ingested algae are only partially utilized [47]. In fact, a fraction of the consumed phytoplankton cells or colonies (e.g., *Microcystis* sp., diatoms, volvocalean, and chlorococcalean green algae) may stay alive after passing through the digestive tract of fish as they are protected either by a mucilaginous envelope or by a thick, cellulose-based cell wall [47,95]. In the present study, 11 *Microcystis* strains were isolated from gut samples and identified as *Microcystis flos-aquae* with a characteristic mucilage envelope that can be the main protecting layer against the extreme gut environment.

In the scientific literature, there is contradictory information on the abundance and composition of cyanobacteria in the alimentary tract of bigheaded carps. On one hand, a study by Ye et al. [96] found cyanobacteria to be predominant in the gut microbiota of bigheaded carp living in different North American rivers, while Li et al. [97] reported the abundance of cyanobacteria was typically low in the intestines of both silver and bighead carp inhabiting Wuhu Lake (China). Based on the gut content metagenome analysis, *Microcystis* was identified as the most abundant cyanobacterial genus detected in the gut of bigheaded carp in Lake Balaton [98].

Beyond the outer polysaccharide layer, it is worth noting the detected peptides in this work, mainly the large number of ANAs and MGs (Table 1 and Table 2) and the above discussed bioactivity. Digestive enzymes’ activity in bigheaded carp species have been investigated in several studies [99,100]. Phosphatases and proteases are principal groups of enzymes for the fish species [101]. “The rapid excretion rate of silver carp would require quick digestion and nutrient uptake of foods to support high growth rates” [102]. Several microorganisms that can play a role in digestion and be responsible for higher levels of the above-mentioned digestive enzymes have been identified in the gut of silver carp [96]. Our suggestion is that the detected MGs and ANAs, as potent protease inhibitors, could modify the digestive capacity by binding directly to the enzymes. However, it is known that most oligopeptides stay in the producing cyanobacteria cell and are only released via cell lysis following cell death, and thus, would only provide protection for the surviving cyanobacterial cells in the gut.

While the traditional approaches of toxin and/or bioactive metabolite research of cyanobacteria have mainly focused on individual peptides, exploring their effects or biosynthesis, our chemotyping study with non-targeted analysis investigated the occurrence of various peptides in *Microcystis* strains that originated from bloom, pelagic plankton samples, and from the gut of a notorious invasive fish species. Except for 13 peptides, all other congeners were detected from viable and cultivated chemotypes originating from bigheaded carps. This finding suggests that the alimentary tract of bigheaded carps is not only a special habitat, but also a supplier for strains that represent the pelagic chemotypes and can initiate blooms in the waterbody. This potentially malicious feature can come from the ability of this fish species to filter plankton efficiently, but a few organisms such as the peptide-producing mucilaginous enveloped cyanobacterial species *M. flos-aquae* are digested improperly or not at all in the digestive system. In addition, several studies have noted that the toxicity of cyanobacteria remained unaffected or even increased after defecation. Kolmakov et al. [103] demonstrated that the physiology of the investigated cyanobacterial species were not suppressed by passing through fish intestines, but rather enhanced when they returned to the water. Kolar et al. [35] also noted that some *Microcystis* cells were not eliminated by the digestive processes of fish species. Lewin et al. [46] suspected that *Microcystis* could survive, and even use the phosphorus in fish guts as nutrients [46].

Wide time interval evacuation rates have been estimated for silver carp at different water temperatures [104]. This is why it is not easy to calculate the retention of viable *Microcystis* cells in the gut. Although it is worth raising the opportunity that bigheaded carps carrying cyanobacterial chemotypes in their guts from one habitat can invade new areas, and that the viable cyanobacterial cells may be released by defecation from fish [32,33].

In our chemotyping study, *Microcystis* strains isolated from the invasive non-native bigheaded carps and their peptide patterns were compared to pelagic and bloom material strains. Our results draw attention to the fact that bigheaded carps not only carry and spread viable, mucilaginous envelope-covered *Microcystis* cells from their alimentary tracts, but harmful cyanobacterial strains can also be found among them according to the chemotypes. 

## 4. Materials and Methods 

### 4.1. Sample Collection and Initial Sample Processing

Bigheaded carp were collected from Lake Balaton (Hungary), which is the largest lake in Central Europe. Its surface area is 596 km^2^, while the average water depth is about 3 m [105]. Bigheaded carps and water samples were collected from the lake in April, May, June, September, and October in 2013.

The local fishery company (Balaton Fish Management Non-Profit Ltd., Hungary) had a permit to harvest fish by nets (including bigheaded carp) from Lake Balaton in 2013 (permit reg. no.: 2013/N000001, issued by the Fisheries Authority of Somogy County, Hungary). The fishery company provided samples to the researchers from their commercial catches. After receiving the samples, researchers of the Balaton Limnological Institute (Center for Ecological Research, Hungarian Academy of Sciences) transported them to the laboratory within 30 min. The Institute has a permit for the delivery and use of fish for scientific purposes (permit reg. no.: VE-I-001/01890-3/2013, issued in 22 August 2013 by the Food-Security and Animal Health Directorate, Governmental Office of Veszprém County, Hungary). Gut content samples were collected aseptically, as described by Görgényi et al. [47]. Subsamples were taken (cc. 5 g) and stored in sterile Eppendorf tubes at 4 °C until laboratory processing, all done within 24 h.

**Figure 6:**
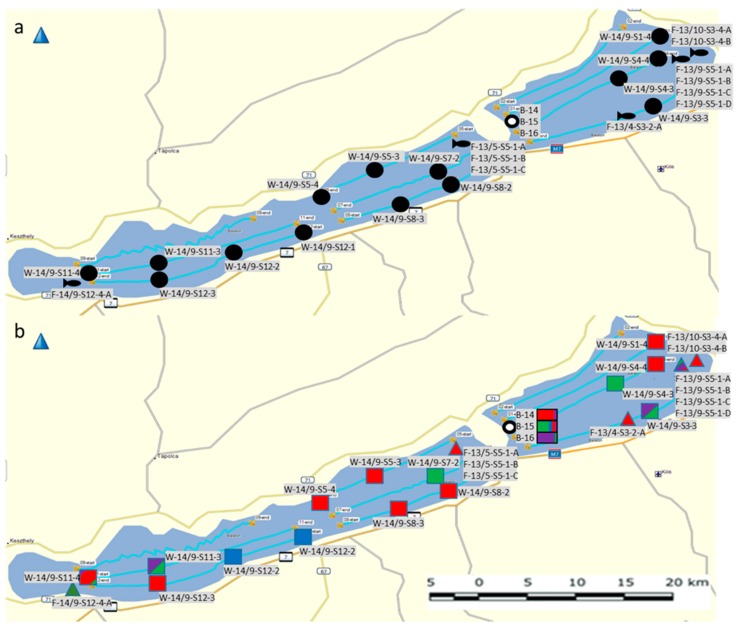
(**a**) Location of the sampling sites for the isolated *Microcystis* strains originating from the pelagic plankton samples: 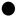; hindgut content of bigheaded carp: 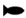; and collected bloom samples: 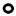. (**b**): Relative abundance of peptide families (microginins: red; anabaenopeptins: green; microcystins: blue; unidentified peptides: magenta) at the sampling sites: Pelagic plankton samples: 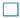; hindgut content of bigheaded carp: 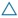, and collected bloom samples: 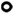.

Water samples for chemical and biological analyses were collected by immersion from the upper water layer at the beginning, one-third, two-third, and ending points of each transect (Figure 6a).

Cyanobacterial bloom samples were collected from blooming waters of *Microcystis* morphotypes during the summer season (July–August) from 2014 to 2016. Twenty-five isolated strains (11 from the hindgut of bigheaded carps and 14 from free living pelagic plankton) and three collected bloom samples were analyzed in this study. Their origin and localization are shown on the map in Figure 6.

Gut content and water samples were incubated in nitrate containing Allen medium [106] for 5 days and the visible *Microcystis* colonies were collected and inoculated in nitrate-containing medium at 26 °C under continuous illumination (100 lux m^−2^s^−1^) for a week. Prior to the molecular analyses, the collected bloom samples and the cyanobacterial strains were studied using light microscopy.

Cells were collected by centrifugation (J-10 rotor of Beckman Avanti J-25; 4500× *g*), lyophilized (Christ Alpha 1-2 LD plus), and then 25 mg of each sample were extracted with 80% methanol. After centrifugation (J-18 rotor of Beckman Avanti J-25; 21,000× *g*), the supernatant was analyzed by HPLC-ESI-MS/MS in positive ion mode.

### 4.2. Identification of Cyanobacterial Peptides

The optimal ESI ionization parameters were as follows: heater temperature, 250 °C; sheath gas, N_2_; flow rate, 10 arbitrary units (arb); aux gas flow rate, 5 arb; spray voltage, 5 kV; capillary temperature, 375 °C; capillary voltage, 35.00 V. Sample measurement was run in positive ion mode (MS). The LC-MS measurements were run on a Thermo Accela HPLC (column: Kinetex XB-C18 100 mm × 2.1 mm × 2.6 µm) attached to a Thermo LTQ XL Linear Ion Trap Mass Spectrometer. Gradient components were (A) water with 0.1% HCOOH and (B) MeCN with 0.1% HCOOH. The time program was 10–70% B: 0–10 min, 70–100% B: 10–11 min, 100% B 11–16 min, 100–10% B: 16–18 min, 10% B: 18–20 min. The injected sample amount was 1.0 µL in all cases.

MS data were processed in Thermo Excalibur version 2.2 SP1.48, and MZmine 2.11 freeware. Identification of secondary metabolites from the MS/MS fragmentation patterns was based on literary data [3,49,51,107].

### 4.3. Statistics

Identified peptides were integrated using targeted peak search in mzMine 2.11 [108]. Thereafter, raw metabolite abundances were scaled and centered separately for each feature in R 3.5.0 [109]. The dataset was hierarchically clustered in both dimensions (samples, metabolites) using the Minkowski distance as the distance measure and Ward’s method. The order of appearance on the presented heatmap’s axes followed that from the clustering. The color strength was proportional to the log10 transformed raw (non-scaled) metabolite abundances, while the color hue was a function of metabolite class: ANA, red; MCY, blue; MG, red; and other peptides, magenta.

The presence of chemotypes was shown by bootstrapped hierarchical clustering analysis of the cyanobacterial lines’ scaled and centered natural compound abundance values using the Minkowski distance and Ward’s hierarchical clustering, bootstrapping *N* = 1 × 10^6^. The calculation was done with the ‘pvclust’ package in R 3.5.2 [110]. To test the statistical significance of chemical patterns and other variables (such as origin, taxonomic position), principal component score values were subjected to the Kruskal–Wallis significance test, with the variable studied being the group-determining factor. This approach allowed for much more statistical power than that of the direct statistical analyses for all metabolites.

### 4.4. Phylogenetic Analysis

To explore the phylogenetic relationships between *Microcystis* strains, amplification and sequence analysis of the 16S–23S internal transcribed spacer region was carried out. DNA amplification was performed by PCR using primers MITS-F (5′-AAGGGAGACCTAATTCVGGT-3′) and MITS-R (5′-TTGCGGTCYTCTTTTTTGGC-3′) [20] in a 2720 Thermal Cycler (Applied Biosystems, Foster City, CA, USA) with the following temperature protocol: Initial denaturation at 95 °C for 5 min, followed by 30 amplification cycles of 30 s at 94 °C, 30 s at 55 °C, and 30 s at 72 °C, followed by a final extension at 72 °C for 3 min. The PCR reaction mixture contained 200 μM of each deoxynucleoside triphosphate, 1 U of LC Taq DNA Polymerase (recombinant) (Fermentas, Vilnius, Lithuania), 1× Taq buffer with (NH_4_)_2_SO_4_ (Fermentas, Vilnius, Lithuania), 2 mM MgCl_2_, 0.3 μM of each primer, and about 20 ng of genomic DNA template in a total volume of 50 μL. PCR products were checked on a 1% agarose gel stained with Eco Safe DNA dye (Pacific Image Electronics, New Taipei City, Taiwan), and visualized using UV excitation.

Sequence analysis of the obtained PCR products was accomplished by Sanger sequencing at LGC Genomics (Queens Road, Teddington, Middlesex, UK), using the MITS-F primer.

The phylogenetic dendrogram of *Microcystis*-related strains was constructed using MEGA7: Molecular Evolutionary Genetics Analysis version 7.0 for bigger datasets [111] software. The evolutionary history was inferred by using the maximum likelihood method based on the Jukes–Cantor model [112]. The tree with the highest log likelihood (−716.14) is shown. The percentage of trees in which the associated taxa clustered together is shown next to the branches. Initial tree(s) for the heuristic search were obtained automatically by applying the Neighbor-Join and BioNJ algorithms to a matrix of pairwise distances estimated using the maximum composite likelihood (MCL) approach, and then by selecting the topology with a superior log likelihood value. A discrete Gamma distribution was used to model the evolutionary rate differences among the sites (five categories (+G, parameter = 0.5472)). The rate variation model allowed for some sites to be evolutionarily invariable ([+I], 43.72% sites). The tree was drawn to scale, with branch lengths measured in the number of substitutions per site. The analysis involved 75 nucleotide sequences. All positions containing gaps and missing data were eliminated. A total of 239 positions were in the final dataset [111].

## Figures and Tables

**Table 1:** Identified microginin type peptides from isolated Microcystis strains. Leucine and isoleucine cannot be distinguished from LC-MS/MS data, these amino acids have been deduced from the nearest literary results.

Compound	[M + H]^+^ *m/z*	RT min	1	2	3	4	5
MG 565	565.5	6.1	Ahda	Thr	Pro	Tyr	
MG 549	549.5	6.2	Ahda	Ala	MePro	Tyr	
MG 579	579.6	6.4	MeAhda	Thr	Pro	Tyr	
MG 579	579.6	6.3	Ahda	Thr	MePro	Tyr	
MG 581	581.5	5.5	Ahda	Thr	Pro	OHTyr	
MG 595	595.5	5.7	Ahda	Thr	MePro	OHTyr	
MG FR3	728.6	6.3	Ahda	Thr	Pro	Tyr	Tyr
MG 712	712.6	6.4	Ahda	Ala	MePro	Tyr	Tyr
MG FR4	742.6	6.6	MeAhda	Thr	Pro	Tyr	Tyr
MG 744	744.6	6	Ahda	Thr	Pro	OHTyr	Tyr
MG 754	754.6	6.7	Ahda	Leu	Pro	Hty	Tyr
MG 607	607.5	6.4	Ahda	Leu	MeVal	Hty	
MG 621	621.6	6.4	MeAhda	Leu	MeVal	Hty	
MG 591	591.5	7.4	MeAhda	Leu	MeVal	Phe	
MG 770	770.6	6.7	Ahda	Leu	MeVal	Hty	Tyr
MG 784	784.6	6.7	MeAhda	Leu	MeVal	Hty	Tyr
MG 798	798.6	7	MeAhda	Leu	MeLeu	Hty	Tyr

**Table 2:** Identified anabaenopeptin type peptides from isolated *Microcystis* strains. Leucine and isoleucine cannot be distinguished from LC-MS/MS data, these amino acids have been deduced from the nearest literary results. Unidentified amino acids were marked with X.

Compound	[M + H]^+^ *m/z*	RT min	1		2	3	4	5	6
ANA B	837.7	5	Arg	CO	Lys	Val	Hty	MeAla	Phe
ANA F	851.7	5.3	Arg	CO	Lys	Ile	Hty	MeAla	Phe
ANA A	844.7	6.6	Tyr	CO	Lys	Val	Hty	MeAla	Phe
OSC Y	858.6	7	Tyr	CO	Lys	Ile	Hty	MeAla	Phe
ANA 828	828.6	7.7	Tyr	CO	Lys	Val	Hph	MeAla	Phe
ANA 842	842.7	8	Tyr	CO	Lys	Ile	Hph	MeAla	Phe
ANA 916	916.9	6.9	Tyr	CO	Lys	Val	Hty	MeHty	Ile
ANA 930	930.6	7.1	Tyr	CO	Lys	Ile	Hty	MeHty	Ile
ANA 852	852.8	7.3	MeHty	CO	Lys	Val	Hty	MeAla	MeLeu
ANA 866	866.7	7.6	MeHty	CO	Lys	Ile	Hty	MeAla	MeLeu
ANA 856	856.7	8.2	MeHty	CO	Lys	Val	Hph	MeAla	Phe
ANA 870	870.7	8.5	MeHty	CO	Lys	Ile	Hph	MeAla	Phe
ANA 872	872.6	7.2	MeHty	CO	Lys	Val	Hty	MeAla	Phe
ANA 886	886.7	7.4	MeHty	CO	Lys	Ile	Hty	MeAla	Phe
ANA 900	900.7	7.8	MeHty	CO	Lys	Ile	Hty	MeAla	MePhe
ANA 914	914.8	8	Tyr	CO	Lys	X	X	X	X
ANA 928	928.7	7.9	Tyr	CO	Lys	X	X	X	X
ANA 892	892.7	6	Tyr	CO	Lys	X	X	X	X
ANA 938	938.5	7.7	Tyr	CO	Lys	X	X	X	X
ANA 860	860.7	6	Tyr	CO	Lys	X	X	X	X
ANA 888	888.7	6.6	MeHty	CO	Lys	X	X	X	X
ANA 902	902.6	6.8	MeHty	CO	Lys	X	X	X	X
ANA 904	904.6	6.6	MeHty	CO	Lys	X	X	X	X
ANA 904	904.7	7.4	MeHty	CO	Lys	X	X	X	X
ANA 934	934.6	6.9	MeHty	CO	Lys	X	X	X	X
ANA 854	854.6	8.1	X	CO	Lys	Ile	Hty	MeAla	Phe
ANA 814	814.7	7.6	X	CO	Lys	Ile	Hty	MeAla	Phe
ANA 888	888.7	6.2	X	CO	Lys	Val	Hty	MeAla	Phe
ANA 920	920.7	8	X	CO	Lys	Ile	Hty	MeAla	Phe
ANA 892	892.6	7.4	X	CO	Lys	X	X	X	X
ANA 984	984.6	7.7	X	CO	Lys	X	X	X	X
ANA 902	902.7	6.5	X	CO	Lys	X	X	X	X
ANA 918	918.6	6.7	X	CO	Lys	X	X	X	X
ANA 905	905.2	6.5	X	CO	Lys	X	X	X	X
ANA 922	922.1	6.6	X	CO	Lys	X	X	X	X
ANA 904	904.7	5.8	X	CO	Lys	X	X	X	X

**Table 3:** Identified microcystin-type peptides from isolated *Microcystis* strains. Leucine and isoleucine cannot be distinguished from LC-MS/MS data, these amino acids have been deduced from the nearest literary results.

Compound	[M + H]^+^ *m/z*	RT min	1	2	3	4	5	6	7
MCY-LW	513.3 ^1^	5.4	Ala	Leu	MeAsp	Trp	Adda	Glu	MeDha
MCY-RR	520.3 ^1^	5.6	Ala	Arg	MeAsp	Arg	Adda	Glu	MeDha
[D-Asp3]MCY-LR	981.9	6.6	Ala	Leu	Asp	Arg	Adda	Glu	MeDha
MCY-LR	995.8	6.8	Ala	Leu	MeAsp	Arg	Adda	Glu	MeDha
MCY-HilR	1009.9	7	Ala	Hil	MeAsp	Arg	Adda	Glu	MeDha
[MeSer7]MCY-LR	1014	6.6	Ala	Leu	MeAsp	Arg	Adda	Glu	MeSer
[Dha7]MCY-FR	1015.8	7	Ala	Phe	MeAsp	Arg	Adda	Glu	Dha
MCY-FR	1029.7	7.1	Ala	Phe	MeAsp	Arg	Adda	Glu	MeDha
[Dha7]MCY-YR	1031.9	6.5	Ala	Tyr	MeAsp	Arg	Adda	Glu	Dha
MCY-YR	1045.5	6.6	Ala	Tyr	MeAsp	Arg	Adda	Glu	MeDha
MCY-(H4)YR	1049.7	6.3	Ala	H_4_Tyr	MeAsp	Arg	Adda	Glu	MeDha
MCY-WR	1068.8	7.2	Ala	Trp	MeAsp	Arg	Adda	Glu	MeDha
[MeSer7]MCY-WR	1086.9	6.9	Ala	Trp	MeAsp	Arg	Adda	Glu	MeSer

^1^ Data are given in [M + 2H]^2+^.

**Table 4:** Unidentified peptides/peptide fragments from isolated *Microcystis* strains. Leucine and isoleucine cannot be distinguished from LC-MS/MS data. Unidentified amino acids were marked with X.

Compound	[M + H]^+^ *m/z*	RT min	*n*	*n* + 1	*n* + 2
PEP 535	535.4	6.7	X	Leu	
PEP 539	539.4	9.4	X	Leu	
PEP 541	541.4	5	X	Phe	
PEP 565	565.5	5	X	MeLeu	
PEP 581	581.5	5.8	X	Tyr	
PEP 593	593.5	6.1	X	Met	MeLeu
PEP 756	756.6	6.8	X	Tyr	Tyr
PEP 593	593.5	6.8	X	Tyr	
PEP 594	594.4	5.2	X	Thr	Leu
PEP 712	712.6	5.4	X	MeLeu	Tyr
PEP 714	714.6	5.2	X	MeLeu	Tyr
PEP 728	728.6	5.4	X	MeLeu	Tyr
PEP 740	740.7	8.4	X	MeLeu	Tyr
PEP 742	742.6	5.5	X	Met	
PEP 744	744.5	5	X	MeLeu	
PEP 714	714.6	5.5	X	MeLeu	Tyr
PEP 728	728.6	5.7	X	MeLeu	Tyr

## Appendix A

**Table A1:** Product ion data for the microginin type peptides. Masses are given in Dalton rounded to the nearest integer. X_1_′ indicates core fragment of Ahda after abstraction of the side chain (m = 58 Da). Leucine and isoleucine cannot be distinguished from the LC-MS/MS data, these amino acids have been deduced from the nearest literary results.

Compound	[M + H]^+^ *m/z*	RT min	Amino Acid Sequence	X_1_-X_2_-X_3_-X_4_	-H_2_O	X_1_’-X_2_-X_3_-X_4_	-H2O	X_1_-X_2_-X_3_	-H_2_O	X_1_’-X_2_-X_3_	-H_2_O	X_1_-X_2_
MG 565	565.5	6.1	Ahda-Thr-Pro-Tyr					384	366	257	239	287
MG 549	549.5	6.2	Ahda-Ala-MePro-Tyr					368	nd	241	nd	257
MG 579	579.6	6.4	MeAhda-Thr-Pro-Tyr					398	380	257	239	301
MG 579	579.6	6.3	Ahda-Thr-MePro-Tyr					398	380	271	253	287
MG 581	581.5	5.5	Ahda-Thr-Pro-OHTyr					384	366	257	239	287
MG 595	595.5	5.7	Ahda-Thr-MePro-OHTyr					398	380	271	253	287
MG FR3	728.6	6.3	Ahda-Thr-Pro-Tyr-Tyr	547	529	420	402	384	366	257	239	287
MG 712	712.6	6.4	Ahda-Ala-MePro-Tyr-Tyr	531	513	404	nd	368	350	241	223	257
MG FR4	742.6	6.6	MeAhda-Thr-Pro-Tyr-Tyr	561	543	420	nd	398	380	257	239	301
MG 744	744.6	6	Ahda-Thr-Pro-OHTyr-Tyr	547	529	nd	nd	384	366	257	239	287
MG 754	754.6	6.7	Ahda-Leu-Pro-Hty-Tyr	573	nd	446	nd	396	nd	269	252	299
MG 607	607.5	6.4	Ahda-Leu-MeVal-Hty					412	394	285	267	299
MG 621	621.6	6.4	MeAhda-Leu-MeVal-Hty					426	408	285	267	313
MG 591	591.5	7.4	MeAhda-Leu-MeVal-Phe					426	408	285	267	313
MG 770	770.6	6.7	Ahda-Leu-MeVal-Hty-Tyr	589	571	462	444	412	394	285	267	299
MG 784	784.6	6.7	MeAhda-Leu-MeVal-Hty-Tyr	603	585	462	nd	426	408	285	267	313
MG 798	798.6	7	Ahda-Leu-MeLeu-Hty-Tyr	617	599	476	458	440	422	299	281	313

**Table A2:** Product ion data for anabaenopeptin type peptides. Masses are given in Dalton rounded to the nearest integer. Leucine and isoleucine cannot be distinguished from the LC-MS/MS data, these amino acids have been deduced from the nearest literary results.

Compound	[M + H]^+^ *m/z*	RT min	Amino Acid Sequence	Ring-CO	Ring	X_6_-X_2_-X_3_-X_4_	X_5_-X_6_-X_2_-X_3_	X_2_-X_3_-X_4_
ANA B	837.7	5	Arg-CO-[Lys-Val-Hty-MeAla-Phe]	663	637	552	460	405
ANA F	851.7	5.3	Arg-CO-[Lys-Ile-Hty-MeAla-Phe]	677	651	566	474	419
ANA A	844.7	6.6	Tyr-CO-[Lys-Val-Hty-MeAla-Phe]	663	637	552	460	405
OSC Y	858.6	7	Tyr-CO-[Lys-Ile-Hty-MeAla-Phe]	677	651	566	474	419
ANA 828	828.6	7.7	Tyr-CO-[Lys-Val-Hph-MeAla-Phe]	647	621	536	460	389
ANA 842	842.7	8	Tyr-CO-[Lys-Ile-Hph-MeAla-Phe]	661	635	550	474	403
ANA 916	916.9	6.9	Tyr-CO-[Lys-Val-Hty-MeHty-Ile]	735	709	518	532	405
ANA 930	930.6	7.1	Tyr-CO-[Lys-Ile-Hty-MeHty-Ile]	749	723	532	546	419
ANA 852	852.8	7.3	Tyr-CO-[Lys-X3-X4-X5-X6]	733	707	nd	nd	nd
ANA 866	866.7	7.6	Tyr-CO-[Lys-X3-X4-X5-X6]	747	721	nd	nd	nd
ANA 856	856.7	8.2	Tyr-CO-[Lys-X3-X4-X5-X6]	711	685	nd	nd	nd
ANA 870	870.7	8.5	Tyr-CO-[Lys-X3-X4-X5-X6]	757	731	nd	nd	nd
ANA 872	872.6	7.2	Tyr-CO-[Lys-X3-X4-X5-X6]	679	653	nd	nd	nd
ANA 886	886.7	7.4	MeHty-CO-[Lys-Val-Hty-MeAla-MeLeu]	643	617	532	440	405
ANA 900	900.7	7.8	MeHty-CO-[Lys-Ile-Hty-MeAla-MeLeu]	657	631	546	454	419
ANA 914	914.8	8	MeHty-CO-[Lys-Val-Hph-MeAla-Phe]	647	621	536	460	389
ANA 928	928.7	7.9	MeHty-CO-[Lys-Ile-Hph-MeAla-Phe]	661	635	550	474	403
ANA 892	892.7	6	MeHty-CO-[Lys-Val-Hty-MeAla-Phe]	663	637	552	460	405
ANA 938	938.5	7.7	MeHty-CO-[Lys-Ile-Hty-MeAla-Phe]	677	651	566	474	419
ANA 860	860.7	6	MeHty-CO-[Lys-Ile-Hty-MeAla-MePhe]	691	665	580	488	419
ANA 888	888.7	6.6	MeHty-CO-[Lys-X3-X4-X5-X6]	679	653	nd	nd	nd
ANA 902	902.6	6.8	MeHty-CO-[Lys-X3-X4-X5-X6]	693	667	nd	nd	nd
ANA 904	904.6	6.6	MeHty-CO-[Lys-X3-X4-X5-X6]	695	669	nd	nd	nd
ANA 904	904.7	7.4	MeHty-CO-[Lys-X3-X4-X5-X6]	695	669	nd	nd	nd
ANA 934	934.6	6.9	MeHty-CO-[Lys-X3-X4-X5-X6]	725	699	nd	nd	nd
ANA 854	854.6	8.1	X1-CO-[Lys-Ile-Hty-MeAla-Phe]	677	651	566	474	419
ANA 814	814.7	7.6	X1-CO-[Lys-Ile-Hty-MeAla-Phe]	677	651	566	474	419
ANA 888	888.7	6.2	X1-CO-[Lys-Val-Hty-MeAla-Phe]	663	637	552	460	405
ANA 920	920.7	8	X1-CO-[Lys-Ile-Hty-MeAla-Phe]	677	651	566	474	419
ANA 892	892.6	7.4	X1-CO-[Lys-X3-X4-X5-X6]	677	651	nd	nd	nd
ANA 984	984.6	7.7	X1-CO-[Lys-X3-X4-X5-X6]	677	651	nd	nd	nd
ANA 902	902.7	6.5	X1-CO-[Lys-X3-X4-X5-X6]	677	651	nd	nd	nd
ANA 918	918.6	6.7	X1-CO-[Lys-X3-X4-X5-X6]	677	651	nd	nd	nd
ANA 905	905.2	6.5	X1-CO-[Lys-X3-X4-X5-X6]	679	653	nd	nd	nd
ANA 922	922.1	6.6	X1-CO-[Lys-X3-X4-X5-X6]	712	686	nd	nd	nd
ANA 904	904.7	5.8	X1-CO-[Lys-X3-X4-X5-X6]	721	695	nd	nd	nd

**Table A3:** Product ion data for microcystin type peptides. Masses are given in Dalton rounded to the nearest integer.

Compound	[M + H]^+^ *m/z*	RT min	Amino Acid Sequence	X_4_-X_5_-X_6_-X_7_-X_1_-X_2_	X_3_-X_4_-X_5_-X_6_	-H_2_O	X_4_-X_5_-X_6_-X_7_	X_4_-X_5_-X_6_	X_7_-X_1_-X_2_-X_3_-X_4_-NH_2_	X_7_-X_1_-X_2_-X_3_-X_4_	X_1_-X_2_-X_3_-X_4_	X_4_-X_5_
MCY-LW	513.3 ^1^	5.4	[Ala-Leu-MeAsp-Trp-Adda-Glu-MeDha]	896	758	740	712	629	600	583	500	500
MCY-RR	520.3 ^1^	5.6	[Ala-Arg-MeAsp-Arg-Adda-Glu-MeDha]	910	728	710	682	599	614	597	514	470
[D-Asp3]MCY-LR	981.9	6.6	[Ala-Leu-Asp-Arg-Adda-Glu-MeDha]	866	714	696	682	599	556	539	456	470
MCY-LR	995.8	6.8	[Ala-Leu-MeAsp-Arg-Adda-Glu-MeDha]	866	728	710	682	599	570	553	470	470
MCY-HilR	1009.9	7	[Ala-HIle-MeAsp-Arg-Adda-Glu-MeDha]	880	728	710	682	599	584	567	484	470
[MeSer7]MCY-LR	1014	6.6	[Ala-Leu-MeAsp-Arg-Adda-Glu-MeSer]	884	728	710	700	599	588	571	470	470
[Dha7]MCY-FR	1015.8	7	[Ala-Phe-MeAsp-Arg-Adda-Glu-Dha]	886	728	710	668	599	590	573	504	470
MCY-FR	1029.7	7.1	[Ala-Phe-MeAsp-Arg-Adda-Glu-MeDha]	900	728	710	682	599	604	587	504	470
[Dha7]MCY-YR	1031.9	6.5	[Ala-Tyr-MeAsp-Arg-Adda-Glu-Dha]	902	728	710	668	599	606	589	520	470
MCY-YR	1045.5	6.6	[Ala-Tyr-MeAsp-Arg-Adda-Glu-MeDha]	916	728	710	682	599	620	603	520	470
MCY-(H4)YR	1049.7	6.3	[Ala-H4Tyr-MeAsp-Arg-Adda-Glu-MeDha]	920	728	710	682	599	624	607	524	470
MCY-WR	1068.8	7.2	[Ala-Trp-MeAsp-Arg-Adda-Glu-MeDha]	939	728	710	682	599	643	626	543	470
[MeSer7]MCY-WR	1086.9	6.9	[Ala-Trp-MeAsp-Arg-Adda-Glu-MeSer]	957	728	710	700	599	661	644	543	470

^1^ Data given in [M + 2H]^2+^.

**Table A4:** Product ion data for unidentified peptide fragments. Masses are given in Dalton rounded to the nearest integer. Leucine and isoleucine cannot be distinguished from the LC-MS/MS data.

Compound	[M + H]^+^ *m/z*	RT min	Amino Acid Sequence	X_n_	X_n+1_
PEP 535	535.4	6.7	X_n_-Leu	404	
PEP 539	539.4	9.4	X_n_-Leu	408	
PEP 541	541.4	5	X_n_-Phe	376	
PEP 565	565.5	5	X_n_-MeLeu	420	
PEP 581	581.5	5.8	X_n_-Tyr	400	
PEP 593	593.5	6.1	X_n_-Met-MeLeu	317	448
PEP 756	756.6	6.8	X_n_-Tyr-Tyr	412	575
PEP 593	593.5	6.8	X_n_-Tyr	412	
PEP 594	594.4	5.2	X_n_ -Thr-Leu	362	463
PEP 712	712.6	5.4	X_n_-MeLeu-Tyr	404	531
PEP 714	714.6	5.2	X_n_-MeLeu-Tyr	406	533
PEP 728	728.6	5.4	X_n_-MeLeu-Tyr	420	547
PEP 740	740.7	8.4	X_n_-MeLeu-Tyr	432	559
PEP 742	742.6	5.5	X_n_-Met	593	
PEP 744	744.5	5	X_n_-MeLeu	599	
PEP 714	714.6	5.5	X_n_-MeLeu-Tyr	406	533
PEP 728	728.6	5.7	X_n_-MeLeu-Tyr	420	547
